# A Pilot Randomized, Controlled Study of Nanocrystalline Silver, Manuka Honey, and Conventional Dressing in Healing Diabetic Foot Ulcer

**DOI:** 10.1155/2017/5294890

**Published:** 2017-01-25

**Authors:** Ka-Kit Tsang, Enid Wai-Yung Kwong, Tony Shing-Shun To, Joanne Wai-Yee Chung, Thomas Kwok-Shing Wong

**Affiliations:** ^1^O&T Department, Queen Elizabeth Hospital, Kowloon, Hong Kong; ^2^Department of Nursing, The Hong Kong Polytechnic University, Kowloon, Hong Kong; ^3^School of Nursing, Tung Wah College, Kowloon, Hong Kong; ^4^Department of Health Technology & Informatics, The Hong Kong Polytechnic University, Kowloon, Hong Kong; ^5^Faculty of Liberal Arts and Social Sciences, The Education University of Hong Kong, Tai Po, Hong Kong; ^6^Ginger Knowledge Transfer and Consultancy Limited, Kowloon, Hong Kong

## Abstract

Nanocrystalline silver (nAg) and Manuka honey (MH) dressing have increasing popularity for treating diabetic foot ulcer (DFU). This study was an open-label randomized controlled trial with three parallel groups' design in examining the preliminary effectiveness of nAg against MH and conventional dressing in healing DFU in terms of ulcer healing, ulcer infection, and inflammation. 31 participants (11 in the nAg group, 10 in the MH group, and 10 in the convention group) diagnosed with type 2 diabetes were enrolled. Wound cleaning, debridement, and topical dressing application were performed according to the group allocation in each visit at weeks 1, 2, 3, 4, 6, 8, 10, and 12. The results found that the proportions of complete ulcer healing were 81.8%, 50%, and 40% in the nAg, MH, and conventional groups, respectively. The ulcer size reduction rate was potentially higher in the nAg group (97.45%) than the MH group (86.21%) and the conventional group (75.17%). In bacteriology, nAg showed a greater rate of microorganism reduction although it was not significant. To conclude, nAg alginate was potentially superior to MH and conventional dressing in healing diabetic foot ulcer in terms of ulcer size reduction rate.

## 1. Introduction

Diabetes mellitus (DM) is a common worldwide problem and diabetic foot ulcer (DFU) is among the most complex and heterogeneous complications in patients with DM [1]. It is estimated that DM affects 8.3% of the global population or 382 million of people [2]. This number continues to grow, making DFU a major public health problem. The cumulative incidences of patients who developed a new appearance of foot ulcer after 1, 3, and 5 years were 27.3%, 57.2%, and 76.4%, respectively, leading to the corresponding reamputation rates of 12.5%, 22.3%, and 47.1% [3].

DFU is also associated with the disruption of normal wound healing mechanism. The persistent inflammation in DFU is likely due to bacterial contamination and subsequent infections [4]. Furthermore, free radicals (superoxide anion and hydroxyl radial) are formed at disproportionately high levels by the formation of advanced glycation end products (AGEP) in people with diabetes [5]. The accumulation of AGEP causes the upregulation of proinflammatory cytokines [such as interleukin-1 (IL-1) and tumor necrosis factor-alpha (TNF-*α*)]. Matrix metalloproteinases (MMPs), which are also produced in wounds, degrade extracellular matrix (ECM) and inhibit growth factors through the formation of reactive oxygen species (ROS) [6]. A topical intervention method that can effectively kill the bacteria and downregulate the MMP and proinflammatory cytokines production is therefore important in the treatment of DFU.

Both paraffin tulle and gauze are conventionally used on DFU. Nevertheless, nanocrystalline silver (nAg) dressing and Manuka honey (MH) dressing have become popular recently in view of the increased risk of multidrug-resistant bacteria in the environment [7]. In vitro evidence indicates that nAg has a unique antibacterial action through anchoring to the bacterial cell wall, causing structural damage altering the membrane permeability [8]. Moreover, recent in vivo studies found that the anti-inflammatory action of nAg could effectively downregulate the level of both MMPs and cytokines [9]. The antibacterial action of MH is mainly based on its acidic nature [10], hyperosmolality to dehydrate bacteria [11], and the phytochemical factor, methylglyoxal (MGO) [12]. Recent in vitro studies further revealed that the cationic and noncationic compounds [13] as well as leptosin [14] contributed to the antibacterial activity in MH. In addition, the flavonoids found in MH could counteract effectively the oxidative stress induced by AGEP in diabetics [15].

Based on the abovementioned evidence, nAg and MH may be able to target the infection and inflammation of DFU. Despite the fact that nAg and MH are increasingly used nowadays, there is limited clinical evidence of high quality to support our practice. Only two low quality randomized controlled trials conducted on MH for DFU are available for the past 10 years [16, 17]. To date, merely one case study series concerning the effects of nAg on DFU was published [18]. It can be considered that MH has stronger evidence than nAg in healing DFU. Most importantly, no comparison study has been conducted to investigate the effectiveness between nAg and MH on DFU. A research gap between the fundamental science and clinical evidence on the DFU healing effects of nAg and MH exists. This was the first pilot randomized controlled trial, which aimed to address this missing link and to investigate the effectiveness of nAg against MH and conventional dressing in healing DFU. We hypothesized that nAg dressing would be more effective than MH dressing and conventional dressing on DFU healing.

## 2. Methods

### 2.1. Study Design

In this open-label prospective pilot randomized control, a study of three parallel groups was designed to compare the clinical effectiveness and the changes in biochemical concentration in wound fluid of nAg against MH dressing and conventional dressing (paraffin tulle) in treating DFU (trial registration: ClinicalTrial.gov NCT02577900). The trial was conducted in compliance with the Declaration of Helsinki. The approval was granted by local university's and hospital's human ethics committees. Informed consent was obtained from every subject before the commencement of the study.

### 2.2. Subjects

The target participants were screened in the orthopedic department of two regional hospitals and one general outpatient clinic (GOPC). Eligible potential subjects were recruited according to the selection criteria after they had been discharged from hospital. All the subjects were referred to and intervened in an outpatient orthopedic nurse-led clinic in an acute regional tertiary hospital of Hong Kong. The inclusion criteria included subjects living in community settings, being type 2 DM patients, age 40 or above with foot ulcer at or below malleolar region, wound size equal to or larger than 1 cm in diameter, and no foreseeable surgery within the 12-week study period through clinician assessment. The exclusion criteria included HbA1c level ≥ 10%; ankle brachial index ≤ 0.4; ulcer with bone or joint exposed; osteomyelitis; severe wound infection [according to the Infectious Diseases Society of America (ISDA) and International Working Group of Diabetic Foot (IWGDF) classification of diabetic foot infection] [19]; known allergy to nAg or MH; known case of venous ulcer, tumor, or autoimmune diseases.

### 2.3. Randomization

The participants were randomized into three groups by online randomization software (http://www.randomization.com). A sequence of randomized group numbers (i.e., 1 to 3) was generated by research assistant A and the randomized group numbers were put into the opaque, sealed envelopes in chronological order. The numbered sealed envelopes were then passed to the first author for enrollment of participants.

### 2.4. Interventions

All participants attended weekly the nurse clinic for follow-up by the first author in the first four weeks and biweekly till the 12th week of the follow-up period. There were eight clinical attendances in total. Regarding the off-loading strategy, customer-made insole (CMI) was provided to participants who had plantar ulcer. Stick and heel walking methods were educated to these participants. Callus debridement was performed by the first author in each clinic visit so as to decrease the local pressure. The ulcer was cleansed and nonviable tissues and biofilm were debrided every time by the first author if necessary after obtaining the verbal consent. All exposed tendons and avascular tissues were also stimulated with needle or blade until they bled. This intervention was used as cellular recruitment to the local area for angiogenesis and granulation formation. Topical dressing was then applied according to the randomization sequence.

The strength of evidence was given by the test treatment of nAg dressing (Acticoat™ absorbent, Smith & Nephew, London, United Kingdom). The active comparator was MH dressing (Medihoney™ gel sheet, Derma Sciences, Toronto, Canada), whereas the control treatment was the paraffin tulle (Jelonet™, Smith & Nephew, London, United Kingdom). As maintained by the prescription of the dressing materials, community nurses or nurses in GOPC undertook simple dressing change. Oral antibiotics were prescribed if the participants had a moderately infected DFU. If the infection was severe, they were recommended to be admitted into hospital and receive intravenous antibiotic injection, and their participation in the study was terminated.

### 2.5. Outcome Measures

The primary outcome of the study was cumulative ulcer healing incidence after 12 weeks of treatment. The secondary outcomes were the ulcer size reduction rate, bacteriology, and clinical signs of wound infection over the 12 weeks as well as the change of TNF-*α*, IL-1*α*, and MMP-9 levels in wound fluid during the first four weeks.

#### 2.5.1. Ulcer Healing

Cumulative ulcer healing incidence was the complete ulcer healing, defined as complete wound closure with smooth epithelial surface. The healing status of ulcer was assessed by research assistant B, who was an experienced registered nurse, in each clinic visit.

#### 2.5.2. Ulcer Size Reduction

During each follow-up visit, a digit wound measurement device (Visitrak digital) was used for wound size measurement by research assistant B. She was unaware of the topical treatment option through waiting outside the clinic until the removal and proper cleansing of the wound had been completed by the first author. In order to ensure the blinding of outcome assessment, thorough cleansing with soap and water was performed before dressing. The residue on the scab was removed with scalpel following the debridement procedure. Follow-up with the participants would be discontinued if the wound was considered completely healed or till the end of the 12-week study period.

#### 2.5.3. Bacteriology

Quantitative tissue swab culture was used as a means to determine the wound bioburden. In order to avoid collecting the biofilm, Levine's technique was used. Wound swab culture was taken on the part of the viable wound bed that has no visible biofilm or nonviable tissue. Wound swab was taken by the first author to each participant for every clinic visit (weeks 1, 2, 3, 4, 6, 8, 10, and 12). The types and quantity of bacteria from the wound swab were recorded.

#### 2.5.4. Clinical Signs of Wound Infection

To date, there is still no specific scoring system to classify DFU infection. In this pilot study, the “IDSA and IWGDF classification of diabetic foot infection” was used. Although the categorization was mostly based on the expert opinions, it was the best available classification system specific to DFU. The infection severity categorization as uninfected, mild, moderate, and severe was performed by research assistant B in every clinic visit.

#### 2.5.5. MMPs and Cytokines Concentration

Wound fluid was collected in the first four weeks by the first author. Ulcer was briefly washed with sterile water before fluid collection. Afterwards, an occlusive dressing was applied over the ulcer. Exudate accumulated under the dressing after 30 minutes to 2 hours was collected with a sterile pipette into 0.4 mL protein Lobind tubes (Eppendorf, Hamburg, Germany). The wound fluid samples were centrifuged at 6,000 revolutions per minute for 30 minutes, aliquoted, and stored at −20°C until further analysis. The protein content of all samples was quantitated and standardized by the BCA Protein assay kit (Pierce Biotechnologies, Rockford, IL, USA) and with bovine serum albumin. The levels of proinflammatory cytokines TNF-*α* and IL-1*α* as well as MMP-9 in wound fluid were determined by commercial enzyme-linked immunosorbent assay human kit (ELISA) according to the manufacture's protocols (Abcam, USA).

### 2.6. Statistical Analysis

All the analyses were carried out according to the intention-to-treat principle. SPSS Statistics for Mac version 22 (SPSS Inc, Chicago, Illinois) was used for data analysis. Comparison was made among groups by Fisher's exact test for nominal data and Kruskal-Wallis test for ordinal and scale data. The complete ulcer healing was compared among groups by Kaplan-Meier estimates. General estimating equation (GEE) was applied to compare the ulcer size reduction rate and bacteriology as well as the wound fluid concentration of MMP9, TNF-*α*, and IL-1*α* among groups. Statistical significance was set at *p* < 0.05 for all tests. Hertzog [20] suggested that the sample size of a pilot study should range from 10 to 40. However, from the clinical experience of the first author, it is quite hard to find eligible participants. Besides, the main objective of this pilot study was to investigate the preliminary effectiveness of nAg dressing on DFU. Therefore, 10 per group were targeted in this pilot study and the total number of 30 participants was planned.

## 3. Results

### 3.1. Baseline Characteristics

**Figure 1:**
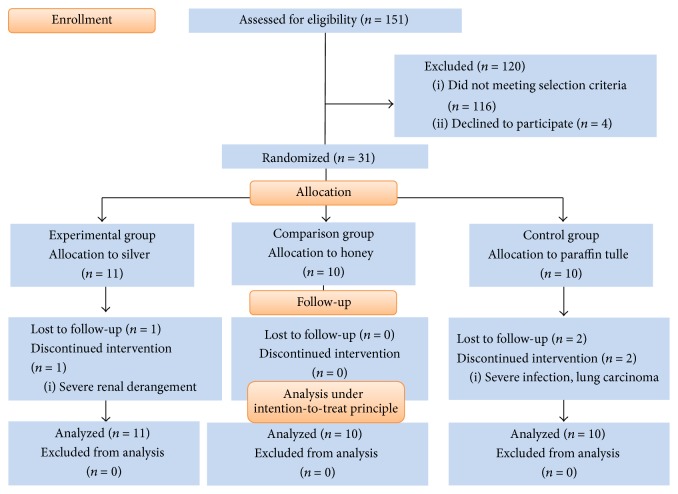
The CONSORT flow diagram.

This study took place from 1 January 2013 to 31 July 2015. Thirty-one subjects (11 in the nAg group, 10 in the MH group, and 10 in the conventional group) were recruited. The CONSORT flow diagram is shown in Figure 1.

The baseline on demographics and risk factors of the subject profiles are presented in Table 1. There were 18 males and 13 females (31 participants in total), 29 of which were recruited from hospitals and 2 from a GOPC. Among all the important parameters affecting DFU healing, there was no statistical difference among groups with *p* values between 0.143 and 0.948.

### 3.2. Cumulative Healing Incidence

#### 3.2.1. Intention-to-Treat Principle

**Figure 2:**
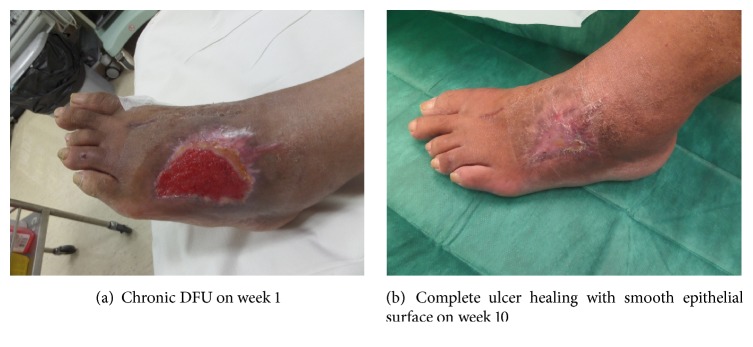
Clinical photos for complete ulcer healing.

**Figure 3:**
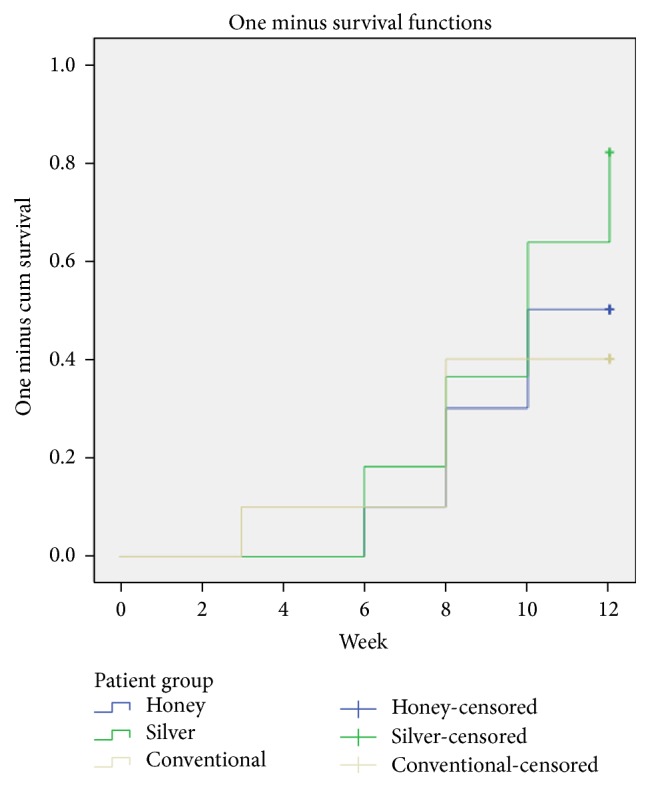
Cumulative healing incidence under the intention-to-treat principle.

The cumulative healing incidence was counted as the incidence of complete ulcer healing in each group (Figure 2). The incidence among groups is shown in Figure 3. In terms of the proportion of complete wound healing at the end of week 12, the nAg group demonstrated the highest proportion (81.8%), followed by the MH group and the conventional group with 50% and 40%, respectively. The overall complete healing was not significant among groups with *p* value 0.267. When the conventional group was used as a reference, the hazard ratio from Cox regression model for the nAg group was 2.179 [95% confidence interval (CI) 0.669–7.906] with *p* value 0.196. In other words, the subjects with DFU in the nAg group were estimated on the average 118% better healing potential at any particular time than those in the convention group. Similarly, the hazard ratio for the MH group was 1.208 (95% CI 0.324–4.504) with reference to the conventional group (*p* = 0.778). The subjects with DFU in the MH group were estimated to have 21% better healing potential than those in the conventional group. However, the wide CI for both hazard ratios, which passed through the null effect of 1, indicated that the differences were not statistically significant.

#### 3.2.2. Per-Protocol Analysis

One subject in the nAg group and two in the conventional groups were prematurely terminated and were excluded. The overall comparison among groups was not significant (*p* = 0.259). When comparing the nAg group and the MH group individually with conventional group, the *p* values were 0.284 and 0.877, respectively. Since similar results were obtained under the intention-to-treat principle and per-protocol analysis, the progress of the subjects with premature termination was similar to that of other subjects in the study.

### 3.3. Ulcer Size Reduction Rate

**Figure 4:**
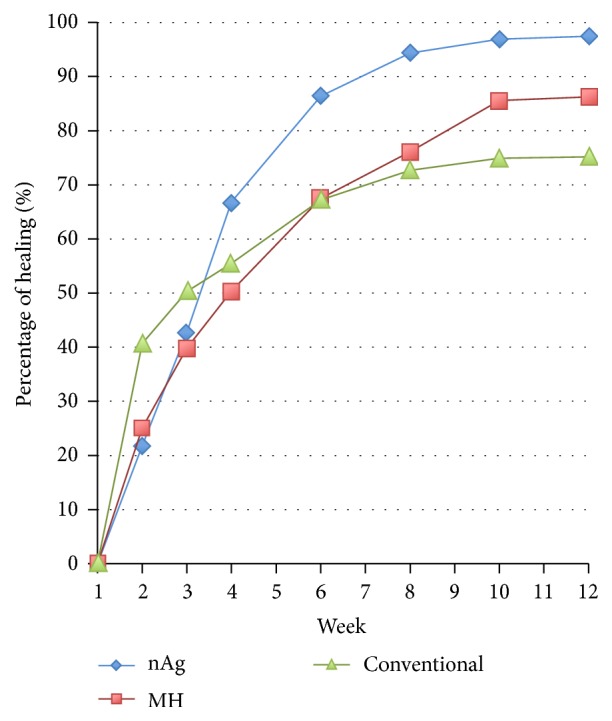
Ulcer size reduction rate in each group.

The ulcer size reduction rate in terms of percentage of area reduction was examined among the three treatment groups. The percentage of area reduction in “week y” was (ulcer area of week 0 – ulcer area of week y)/ulcer area of week 0 × 100%. The mean ulcer size reduction rate in each group is shown in Figure 4.

The nAg group (97.45%) had a higher reduction rate than the MH group (86.24%) and the conventional group (76.91%). The GEE was used to test whether any interaction effect among the groups would be found. The results showed that there was an interaction effect among the three groups with *p* < 0.0005. This means that the ulcer size reduction rate in the nAg group was potentially higher than that in the MH group and the conventional group.

When the gradients of the three lines of the respective groups were compared with the conventional group set as the reference, the slopes of nAg and MH were 20.573 and 9.337, respectively. The positive values of the slopes in the nAg and MH groups imply an increasing trend when compared with the conventional group, with *p* values of 0.011 and 0.311, respectively. This indicated that the ulcer reduction rate of the nAg group was potentially higher than that of the conventional group. With the MH group acting as a reference, the nAg group had a potentially higher ulcer reduction rate than the MH group (*p* < 0.0005) (Table 2). As a whole, the nAg group had a higher ulcer size reduction rate than the MH group and the conventional group.

### 3.4. Bacteriology

**Figure 5:**
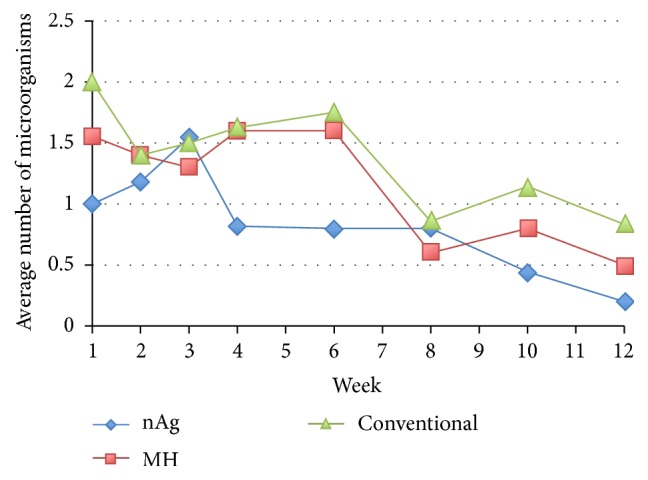
Average number of microorganisms among groups.

In week 1, the numbers of species of microorganism of the participants were 1.00 (SD = 1.00), 1.56 (SD = 1.59), and 2.00 (SD = 1.25) in the nAg group, MH group, and conventional group, respectively. There was no significant difference in the number of species of microorganisms in week 1 among the three groups (*p* = 0.182). The numbers of species found among the groups all followed a decreasing trend over the 12-week study period. The decreasing trend in all three groups is shown in Figure 5. Examining the rate of decrease in the species of microorganisms among groups using the GEE showed that there was a significant difference in the overall interaction effect of “group and week” among groups, with *p* value 0.003.

When the interaction effect between groups was compared, the estimated marginal mean number of microorganisms in the nAg group was the lowest (0.86). The number of microorganisms in the conventional group was the highest (1.36) and that in the MH group was 1.17. The difference in means among the groups was not significant (*p* = 0.486). Although the nAg group did not show a significant decrease in bacteriology as compared with the MH group and the conventional group, it showed the greatest decreasing trend in the number of species of microorganisms.

The most common types of microorganisms in nAg group were* Klebsiella* and* Pseudomonas aeruginosa*. The average quantity of these two microorganisms was “heavy growth” by using the four-point scale of semiquantification surface swab count. On the other hand, the most common type of microorganism was* Klebsiella* in MH group. The corresponding quantification wound swab surface count was “heavy growth.” In the conventional group, the most common type of microorganism was coagulase negative staphylococcus. The respective concentration on the wound bed was “moderate growth.”

Besides, the presence of biofilm was another clinical parameter for the bacterial concentration on wound surface. There were eight observation points throughout the 12 weeks of the study period: weeks 1, 2, 3, 4, 6, 8, 10, and 12. At each time point, the presence or absence of biofilm was compared among groups using Fisher's exact test. The results are shown in Table 3 and found that there were no significant differences at each time point among groups throughout the 12-week study period.

### 3.5. Clinical Signs of Wound Infection

At each observation point, the presence or absence of clinical signs of infection was compared among groups using Fisher's exact test. The results are demonstrated in Table 4, which shows that there were no significant differences at different observation points among the three groups throughout the 12-week study period.

### 3.6. Concentration Profile of Total Protein, MMP-9, TNF-*α*, and IL-1*α* in Wound Fluid

In week 1, the concentrations of total protein, MMP-9, TNF-*α*, and IL-1*α* in wound fluid had no significant differences among groups with *p* value ranging between 0.804, 0.053, 0.209, and 0.184, respectively.

**Figure 6:**
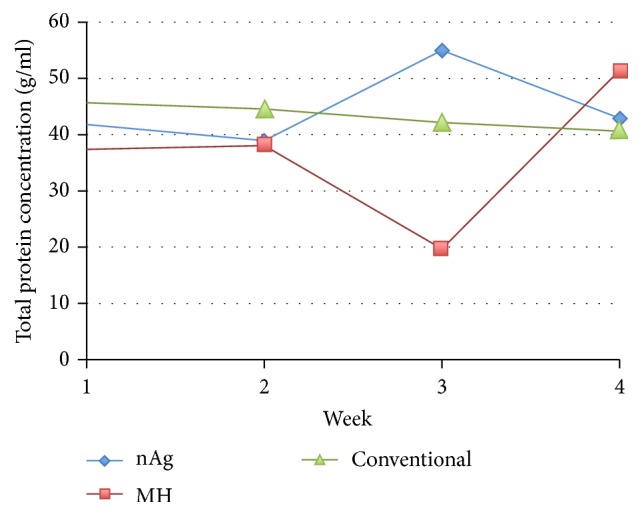
Mean concentration profile of total protein among the three groups.

The change in mean total protein concentration was analyzed among the groups. There was an up and down trend in the nAg and MH groups. Total protein decreased steadily in the conventional group (Figure 6). When examining changes in the concentration among groups over the first 4 weeks, the GEE indicated that there was no significant difference in the entire overall “week and group” effect, with *p* value 0.081, nor was there any interaction effect among (*p* = 0.782) or within groups (*p* = 0.992) over time.

**Figure 7:**
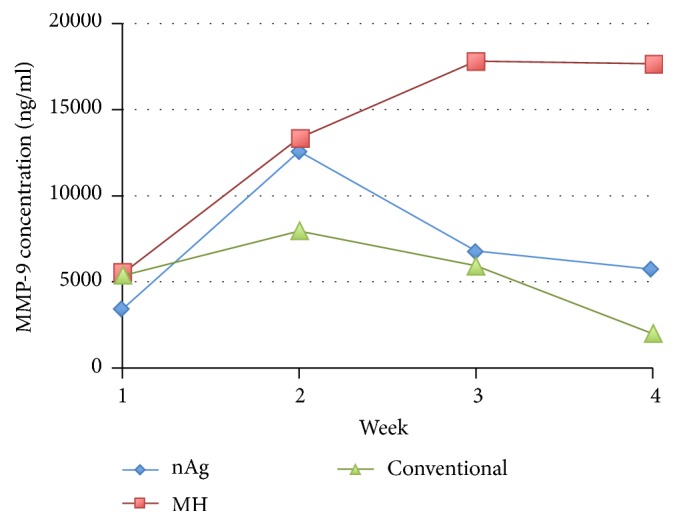
Mean concentration profile of MMP-9 among the three groups.

The change in mean MMP-9 concentration among the groups over the first 4 weeks was considerable as shown in Figure 7. An upward trend in the MH group was noted while the nAg and conventional groups reported a nonlinear trend.

Using GEE analysis model, an overall interaction effect among the groups was observed with *p* value < 0.0005. When the gradients of the three lines of the respective groups were compared with the conventional group set as the reference, the slopes of nAg and MH were 1.263 and 1.507, respectively. The positive values of the slope in the nAg and MH groups implied an increasing trend when compared with the conventional group, with *p* values of 0.027 and <0.0005, respectively. This showed that there was a significant increasing trend between the nAg/MH groups and the conventional groups. When the MH group was taken as the reference, there was no significant difference between it and the nAg group (*p* = 0.089) (Table 5).

**Figure 8:**
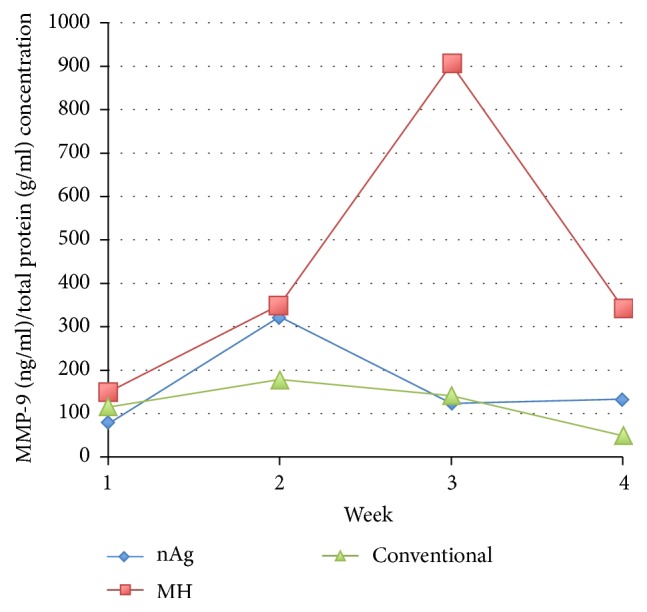
Mean concentration profile of the ratio of MMP-9 and total protein among the three groups.

When the ratio of MMP-9 and total protein was compared among groups over the first 4 weeks, there was still no obvious trend found in each group (Figure 8). The GEE model showed that there was no overall interaction effect among groups over time (*p* = 0.753).

**Figure 9:**
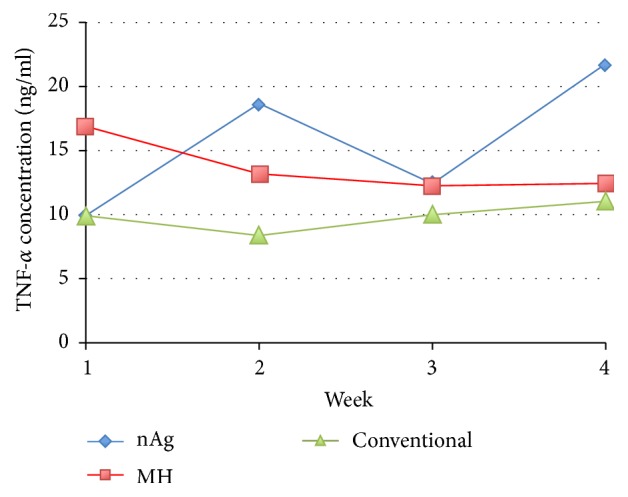
Mean concentration profile of TNF-*α* among the three groups.

Similar to MMP-9, the change in mean TNF-*α* concentration in wound fluid was extensive within each group over the 4-week period. As shown in Figure 9, TNF-*α* in the MH group decreased whereas that in the nAg group, on the contrary, increased. By contrast, TNF-*α* in the conventional group remained more or less constant.

**Figure 10:**
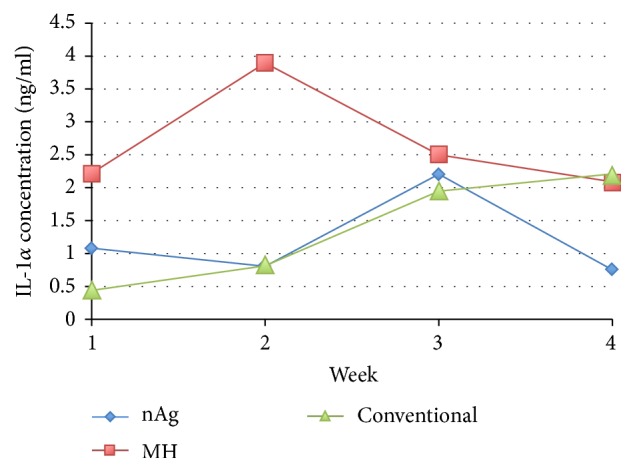
Mean concentration profile of IL-1*α* among the three groups.

On interaction effect examination, there was no overall “week and group” effect (*p* = 0.061). For IL-1*α*, the variations in level within the groups were high. The trends in the change in mean IL-1*α* concentration differed in the three groups, the tendency of which was found similar in the case of MMP-9 and TNF-*α* (Figure 10). By using the GEE model to assess the interaction effect, no overall “week and group” interaction effect was found among the groups with *p* value 0.177.

### 3.7. Use of Antibiotics

The use of antibiotics was a confounder in affecting changes in bacteriology and the clinical signs of wound infection on DFU. Hence, it would affect ulcer healing. Only one participant with severe wound infection in the control group needed intravenous antibiotics during the study period. He was discontinued with the topical intervention, prematurely terminated from the study, and admitted to the hospital. There were totally 30 episodes of participants taking oral antibiotics in the study period: 12 in the nAg group, 11 in the MH group, and 7 in the conventional group. In each clinic visit, one participant taking oral antibiotics was counted as one episode. Among these episodes, around half involved participants continuing taking antibiotics upon discharge from hospital. Only five episodes involved the participants commencing oral antibiotics during the study period. Every single time point was compared among groups using Fisher's exact test. There were no significant differences among the three groups. The *p* values were between 0.174 and >0.9999. The result revealed that there were no significant differences in the distribution of antibiotic use among the three groups throughout the 12-week study period. This implies that the confounder “use of oral antibiotics” did not affect the comparison of ulcer healing among groups.

### 3.8. Adverse Event

In addition, there were six episodes of adverse events occurring during the study period. Four were in the conventional group, and there was one in each of the MH and the nAg groups. Among all the adverse events, four of them were prescribed antibiotics (Table 6). By using the Chi-square, there was no significant difference in the adverse events among groups (*p* = 0.54). The details of the adverse events and interventions were shown in Table 6.

### 3.9. Cost-Effectiveness Analysis

The prices of nAg, MH, and paraffin tulle dressings varied in Hong Kong. The prices of nAg dressing (Acticoat absorbent), MH dressing (Manuka™ honey gel sheet), and paraffin tulle (Jelonet) of 10 × 12.5 cm were (in Hong Kong dollars) $158.00, $225.00, and $13.75 per piece, respectively. The prices of Acticoat absorbent and Manuka honey gel sheet were 11.5 times and 16.4 times higher than that of paraffin tulle dressing. When we compared the ulcer size reduction rate in week 12, the nAg group was 97.25%. The MH group and the conventional group were 86.24% and 76.91%, respectively. However, the clinical effectiveness of Acticoat absorbent and that of Manuka honey gel sheet were only 1.3 times and 1.1 times better than that of paraffin tulle dressing. In this study, all of the participants were outpatients who received daily dressing. The costs for dressing by community nurse and O&T nurse clinic attendances were the same. Therefore, if specialized nursing care including showering before dressing and debridement was performed, paraffin tulle dressing was recommended from the point of view of cost-effectiveness according to the preliminary data. Importantly, further solid recommendation can be made until more subjects are recruited in the future study.

## 4. Discussion

### 4.1. Cumulative Healing Incidence

To the best of our knowledge, this is not only the first pilot randomized controlled trial to compare the effect of nAg dressing, MH dressing, and conventional dressing on DFU, but also the first attempt to examine the changes in MMP and cytokines levels in wound fluid among different treatment groups. In this pilot study, the proportion of healing in the nAg group was higher than that in the MH group and the conventional group for more than 30% at the end of the 12-week study period. Yet, the difference did not reach statistical significance. From the published randomized controlled trials, there was no direct comparison of nAg against MH on DFU. Fries et al. [21] compared the effectiveness of nAg dressing against plain gauze in the postdebridement of military wounds. From the figures reported in the paper, it was found that the days of wound healing in the nAg group (about 21 days) were slightly longer than those of the control group (about 19 days) although the result had no significant difference.

The findings from Fries et al.'s study did not support the present study. The reason for the differences in the present study and Fries et al.'s study may be due to the differences in wound types. In the present study, most of the ulcers were chronic or acute on chronic wounds, while the ulcers in Fries et al.'s study were acute wounds. This may be the difference in the degree of inflammation. The DFU involved in the present study had mild to moderate infection. All the DFU had 1 to 3 types of microorganism cultured in the first week. However, in Fries et al.'s study, there were 58% and 37% yielded negative culture of microorganism on the intervention and control groups, respectively. The low bacterial loading in Fries et al.'s study might decrease the efficiency of antibacterial effect in nAg dressing.

In addition, Gottrup et al. [22] reported on using collagen silver against standard care in DFU. They found that 52% were healed in the collagen silver group while 31% were healed in the control group. However, the topical dressing in the treatment group contained collagen and silver. Both collagen and silver contributed to the DFU healing simultaneously. Although our present findings were similar to Gottrup et al.'s study, it was impossible to identify the single effect of silver. The study results from the two studies made it difficult to perform a direct comparison.

Kamaratos et al. [17] compared the MH and standard care on DFU. The result was similar to our study. They found that 97% were healed in MH group and 90% under conventional care. There was no noticeable difference between the groups. The proportion of healing in both groups in their study was higher than that of the present study. This can be explained by the fact that they excluded subjects with ABI < 0.9 so the ulcers had a better blood perfusion, which may result in higher proportion of complete wound healing. The selected ulcers were less severe than our study's. Al Saeed [16] performed another comparison study between MH dressing and conventional dressing on DFU. The author illustrated that MH was a superior topical intervention to the conventional dressing. The proportion of healing in MH group and control group was 61.3% versus 11.5% at six-week intervals. The proportion of healing in Al Saeed's study was much higher than that reported in the present study. It might be due to the reason that surgical interventions, including toe amputation, were performed to all participants during the study period. This may facilitate the ulcer healing in Al Saeed's study.

### 4.2. Ulcer Size Reduction Rate

In the present study, the percentage of reduction in ulcer size was potentially higher in the nAg group when compared with the MH group and the conventional dressing group at the end of week 12. Ulcer size reduction is a sign of ulcer healing. It was a common study outcome to indicate wound healing in wound care research. In the present study, almost all the DFUs had mild to moderate wound infection in the 1st week of the study, so the potency of antibacterial effect became important for healing in this study. Kwakman et al. [13] found that the antibacterial effect of MH was onset slower than nAg. On the other hand, the exact mechanisms of the anti-inflammatory effect of MH and nAg were not entirely clear and their relative potency was unknown. The conventional dressing had neither antibacterial nor anti-inflammatory effect. That was the reason for the result in this study that the nAg dressing was superior to the MH dressing and the conventional dressing in ulcer size reduction.

Only a few studies ever compared the ulcer size reduction rate between groups directly. The strength of this pilot study was the use of repeated measures to compare the DFU size reduction rate longitudinally among groups. Miller et al. [23] compared the effectiveness of nAg on venous ulcer and had similar findings to ours. They revealed that the proportion of healing between the nAg group and the cadexomer iodine group was not statistically different within 12 weeks of time but the wound healing rate in the first two weeks was faster in the nAg group on larger, older, and more exuding wounds by using the linear mixed model for comparison. This may be due to the fact that large wounds with more exudate usually have higher bacterial concentration. For this type of wound, the antibacterial action of nAg became more important and significant difference was found in this type of wound.

Regarding the small sample size in this study, the preliminary findings could not draw a solid conclusion but it served as a useful reference as a pilot. The preliminary data suggested that the nAg dressing was potentially better than the MH and conventional dressings in DFU healing in terms of wound size reduction rate. It also revealed that the complete wound healing was also potentially higher in the nAg dressing than the MH and conventional dressings despite the fact that it was not significant. This may be because the sample size was not large enough to differentiate the differences in the cumulative healing incidence but it was sufficient to work out the ulcer size reduction rate among groups. Importantly, the soap cleansing and repeated sharp debridement were given to all participants and this may further reduce the detectable differences among groups. It was because soap cleansing and sharp debridement removed nonviable tissues, debris, and biofilms [24], which could help in ulcer healing.

### 4.3. Changes of Bacteriology

Examining the changes in bacteriology should include the types of species of microorganism and the corresponding quantity. Due to the small sample size in this pilot study, only the types of species of microorganism were included for analysis. The decrease in the number of species of microorganism was not different in the three groups but the estimated marginal mean indicated the least bacteria in the nAg dressing group followed by the MH and the conventional dressing groups. Although MH has MGO [12] that is responsible for antibacterial function, the hyperosmolarity [11] is probably the greatest effect that is bacteriostatic rather than bactericidal. In addition, the antibacterial effect of nAg is much faster than that of MH [13]. These are the possible reasons for the change in bacteriology in descending order: nAg dressing, MH dressing, and conventional dressing. However, the specialized nursing intervention of cleansing the ulcer by soap and water before dressing in all participants would reduce the bacteria on the ulcer surface by the surfactant effect. This may further decrease the differences of bacteriology among groups.

This result was supported by those reported in the in vitro and clinical studies. In the in vitro study, Bradshaw [25] found that there was no significant difference between nAg and MH dressings on common wound pathogen including* E. coli*,* S. aureus*, and* P. aeruginosa*. Kamaratos et al.'s [17] clinical study revealed that there was no significant difference in presence of sterile DFUs between their MH and conventional dressing groups with 63 patients. However, 78.13% of ulcers became sterile in the MH dressing group versus 35.5% in the conventional dressing group during the first week. The corresponding percentages for weeks 2, 4, and 6 were 15.62%, 38.7%, and 6.25% in the MH group and 12.9%, 0%, and 12.9% for weeks 2, 4, and 6 in the conventional dressing group. As a whole, the present study and the previous studies showed that the participants with MH dressing or nAg dressing had an observable lower number in bacteria but did not reach the statistical significance.

Throughout the study period, there was no significant difference in the presence of biofilm among the three groups from week 1 to week 12 even though nAg dressing and MH dressings had the antibacterial effect. The possible reason for the insignificant result was probably due to the effect on debridement on every clinical visit since debridement was the most simple and effective method for biofilm removal [26].

### 4.4. Clinical Signs of Wound Infection

In the present study, there was no significant difference in the signs of wound infection among the three groups from week 1 to week 12 although the nAg and MH dressings had the antibacterial effects. As a whole, the percentage of clinical signs of wound infection was also not significant among groups over the 12 weeks. The result from our study was not supported by the published literature. Gottrup et al. [22] compared the silver collagen against the control group with the local standard dressings on DFU and found that the silver collagen group had no signs of infection, while the control group had 31% of infection. Al Saeed [16] compared the effects of MH dressing versus tulle gras on DFU. It showed that MH dressing combined with surgery had an obvious shorter time to eradicate infection than the control group with conventional dressing combined with surgery.

The present preliminary result on clinical signs of wound infection was different from these previous studies. The possible reasons for this difference are as follows. Firstly, specialized nursing education was given to each of the participants. They were required to perform showering with running water before dressing. The specialized nursing intervention on debridement could also remove the biofilm and the nonviable tissues. These procedures could further decrease the bacterial concentration on the wound bed in the three groups. Secondly, regardless of the wound types, the possible reason was the small effect size of the antibacterial effect between the nAg/MH dressing, nAg/conventional dressing, and MH/conventional dressing so that the small sample size could not detect the difference statistically.

### 4.5. The Change in Concentration of MMP-9 and Ulcer Healing

As shown in the result, the trend of MMP-9 concentration in the nAg, MH, and conventional dressing groups fluctuated. The trend of MMP-9 concentration in the three groups was not along with DFU healing in the 4-week interval. The gradient analysis indicated that the increasing rate of MMP-9 concentration in the nAg and MH dressing groups was potentially higher than the conventional group. In combination with these results, it implied that the nAg and MH dressing groups had poor healing as compared with the conventional dressing group because the activities of MMP-9 declined when wound started to heal [27, 28].

However, as reported above, the nAg group had better ulcer healing in terms of higher reduction rate of ulcer size than the conventional dressing group. Therefore, it was found in this study that the change of concentration level of MMP-9 did not align with the reduction rate of DFU size, which was different from the results of the previous studies. According to the published literature, it was found that the change in concentration of MMP-9 was negatively correlated with the wound healing status [27, 28]. The concentration of MMP-9 declined in wound healing in progress.

Indeed, MMP-9 is a well-recognized MMP that plays an important role in normal healing [25] and appears to be the major protease responsible for matrix degradation in chronic wound fluid [29]. MMP-9 is produced by a number of inflammatory cells, including neutrophil, macrophage, and monocyte. In the proliferative phase of wound healing, their activities decline [30]. This is the reason why MMP-9 decreases in the normal wound healing process.

The possible reasons for the difference between the present pilot study and the previous studies were that some of the DFUs of certain participants in the present study were in a rather acute stage when compared with the above studies and had surgeries prior to the recruitment in the present study. Theoretically, the debridement procedure in the operation theatre would cause wound bleeding and turn the ulcer into an acute stage. In addition, compared with the DFUs in Cullen et al.'s study [31], which were noninfected, the DFUs in the present study had different degrees of infection. The bacteria toxin recruited the inflammatory cells for the production of proteases [27]. It would increase the proteases level and the levels were not downregulated during the inflammatory and infected stage regardless of the use of topical dressing material. Therefore, in our study, the concentration of MMP-9 in both nAg and MH dressing groups did not decrease.

### 4.6. The Change in Concentration of Cytokines TNF-*α* and IL-1*α* and Ulcer Healing

It was found in the present study that the trends of cytokines TNF-*α* and IL-1*α* concentrations in the three groups were up and down in the 4-week interval. There was no obvious increasing or decreasing trend. This tendency was the same as the trend of total protein and MMP-9 concentration. The interaction effect was not statistically significant. Interestingly, TNF-*α* is a powerful inducer of MMP-9 [32]. From the study result, we found that the mean concentration of MMP-9 was higher in the MH group than the nAg group. However, the concentration of TNF-*α* was vice versa between both groups. The possible explanation may be due to other unknown factors in affecting the relationship between MMP-9 and TNF-*α*. Importantly, it needs to be further explored in the future studies.

Indeed, both TNF-*α* and IL-1 are mainly secreted by neutrophils and macrophages. TNF-*α* helps in collagen synthesis, while IL-1 is used to recruit fibroblasts and keratinocytes and in collagen synthesis [33]. Macrophages and neutrophils are the dominant cells in the inflammatory stage of wound healing and their activities are minimized in the proliferative stage [34]. As a consequence, the levels of IL-1 and TNF-*α* decrease as the wound starts to heal. Trengove et al. [35] studied the mitogenic activity of healing and nonhealing ulcer. They found that the levels of proinflammatory cytokines TNF-*α* and IL-1 were downregulated during the healing process. Yussof et al. [33] suggested that cytokines including TNF-*α* and IL-1 hold the most potential to predict wound healing.

### 4.7. Possible Reasons for the Inconsistency between the Results of Ulcer Healing and the Change in Concentration of Biomarkers

We could speculate several possible reasons for the inconsistent findings between the laboratory and clinical observations. Firstly, most of the previous researches in this area mainly focused on the chronic nonhealing and noninfected wounds. Some even compared the acute wound fluid with the chronic wound fluid [27, 35, 36]. One of the studies focused on the noninfected ulcer [31] or chronic ulcer only [30]. In the present pilot study, chronicity of ulcers varied. It is noteworthy that all of the DFUs in this study were in the healing stage during the study period.

Secondly, as mentioned before, soap and water cleansing before dressing would decrease the bacterial loading and it would affect the MMPs and cytokines levels since the bacteria were an important factor to recruit the inflammatory cells for secreting these biomarkers. Thirdly, serial sharp debridement was performed in every follow-up during the study period. There were different degrees of bleeding after the debridement procedure. Apart from debridement, the avascular tissue was stimulated to bleed because most of the ulcers involved in this study had tendon and fascia exposed. As a result, acute minor trauma was performed periodically during the study period. The related interventions produced new wounds in order to attract neutrophils and fibroblasts to migrate to the local area. The cells were being stimulated to produce MMPs and cytokines [33]. Although the above interventions produced counterbalance effect, these interventions might be the important cofounders affecting the levels of MMPs and cytokines.

Another possible reason is that these studies categorized the chronic wounds into healing and nonhealing groups and tested the differences of the biomarkers concentration between groups [37–39]. However, in the present study, the concentration of the biomarkers in wound fluid was the laboratory outcome while complete wound healing and ulcer size reduction were the clinical outcomes. The two types of outcomes were triangulated to evaluate the effectiveness of the nAg dressing against the MH and conventional dressings. Therefore, the purpose of this present study in this area was different from that of the previous studies [37–39]. In our pilot study, each group contained DFU relatively “good response” and “poor response” to the topical dressings; this may counteract the mean concentration of those biomarkers in each group. This was the possible reason why different results were obtained comparing with the published studies.

To the best of our knowledge, this was the first attempt to compare the serial changes of these biomarkers with the wound healing status. The result from the present study in this area was preliminary, so further studies with different topical interventions to confirm the result were recommended. Importantly, the investigation into the changes of biomarkers in wound fluid is increasingly popular for the prediction of wound healing. Therefore, there is a need for further researches to clarify any confounder to affect the concentration of biomarkers; otherwise, the creditability and effectiveness of measuring the biomarkers would be in question.

Importantly, the study results contributed to an evidence-based practice for DFU management. Traditionally, the use of dressing materials is mostly based on the clinical judgment of front-line health care providers. Now, this study has provided evidence on the nAg dressing that is potentially better in ulcer healing in terms of ulcer size reduction than the MH and conventional dressings, allowing professionals to select this effective dressing to promote DFU healing in their daily practice.

## 5. Conclusion

In the present study, the baseline characteristics were comparable among groups. All subjects received debridement from the same investigator using standard off-loading techniques. According to the preliminary findings, the authors concluded that the nAg dressing was potentially effective, while MH was only potentially, marginally effective in treating DFU. The preliminary findings in this study suggested a full study should be valid for and worth further investigations. In addition, the acute healing ulcer and ulcer having received serial debridement may not be suitable for comparing the chosen biomarkers to assess the healing status.

The small sample size was the major limitation in our study. The types of ulcers and the stages of acuteness were different among the three groups although there was no overall significant difference. This may create possible random bias in our samples. Nonetheless, the positive preliminary result on the ulcer reduction rate indicates that a clinic trial of larger scale is warranted in order to support the present findings. Another limitation was the insufficient censor points to examine the complete ulcer healing among groups. In our study, there were only nine observations throughout the 12 weeks and it may not be accurate enough to assess the actual healing time. This may explain why there was no significant difference detected among groups although the nAg group saw an obvious, higher cumulative healing incidence. Besides, the small sample size made it impossible to perform detailed bacteriology analysis on both number and types of species of bacteria although nAg showed the greatest decreasing trend in the number of species of microorganism. Finally, we demonstrated that nAg dressing is potentially better for DFU healing in terms of the ulcer size reduction rate as compared with MH and conventional dressings. The results of this clinical randomized trial shed new light on the practice, especially for clinicians, on the use of nAg dressing in DFU. It can also serve as a foundation for future's study on nAg and the related molecular biology on DFU healing.

## Figures and Tables

**Table 1:** Comparison of baseline information on demographics and risk factors among groups.

	nAg (*n* = 11)	MH (*n* = 10)	Conventional (*n* = 10)	*p* value	Significance
Origin of participants					
Hospital A	9	7	8	0.134^a^	NS^*∗*^
Hospital B	2	3	0		
GOPC	0	0	2		
*Personal factors*					
Gender					
M	7	4	7	0.433^a^	NS^*∗*^
F	4	6	3		
Age (years) [mean (SD)]	63.36 (11.31)	65.60 (11.42)	66.1 (12.31)	0.948^b^	NS^*∗*^
Serum albumin level (mmol/ L) [mean (SD)]	32.55 (6.99)	33.80 (4.89)	37.40 (4.84)	0.147^b^	NS^*∗*^
Ambulatory status					
Ambulatory	11	10	10	NA	NA^*£*^
*Disease factors*					
Duration of diabetes (years) [mean (SD)]	14.82 (10.44)	13.30 (9.63)	15.20 (9.88)	0.921^b^	NS^*∗*^
HbA1c level (mmol/ L) [mean (SD)]	8.27 (1.32)	8.30 (2.26)	7.59 (0.99)	0.574^b^	NS^*∗*^
Ankle brachial index (ABI) [mean (SD)]	1.06 (0.20)	1.03 (0.24)	1.13 (0.21)	0.944^b^	NS^*∗*^
Heart disease					
Yes	4	5	6	0.604^a^	NS^*∗*^
No	7	5	4		
PAD					
Yes	1	0	1	>0.9999^a^	NS^*∗*^
No	10	10	9		
*Local ulcer factors*					
Ulcer location on foot					
Toe amputation	7	2	2	0.391^a^	NS^*∗*^
Dorsum	1	2	1		
Plantar	0	1	3		
Plantar to dorsum	1	0	0		
Medial malleolus	0	1	1		
Anteromedial ankle	1	2	0		
Lateral malleolus	1	1	2		
Heel	0	1	1		
Ulcer chronicity^∧^					
Acute	7	5	6	0.901^a^	NS^*∗*^
Chronic	4	5	4		
Ulcer duration (weeks) [mean (SD)]	11.45 (6.67)	12.80 (10.54)	14.70 (8.12)	0.401^b^	NS^*∗*^
Ulcer size (cm^2^) [mean (SD)]	8.68 (6.84)	10.98 (8.03)	8.28 (7.27)	0.495^b^	NS^*∗*^
University of Texas (UT) classification Ulcer grade (depth)					
0	0	1	1	0.867^a^	NS^*∗*^
1	5	5	5		
2	6	4	4		
3	0	0	0		
SWESS (score of 0–30) [mean (SD)]	13.27 (2.15)	14.40 (1.90)	12.27 (2.31)	0.195^b^	NS^*∗*^
Clinical signs of infection					
None	7	3	6	0.498^a^	NS^*∗*^
Mild	3	6	3		
Moderate	1	1	1		

^a^Fisher's exact test.

^b^Kruskal-Wallis test.

^*∗*^Not significant.

^*£*^Not available.

Significant (*p* ≤ 0.05).

^∧^Chronic wound was defined as wound duration ≥ 12 weeks.

**Table 2:** Gradients of the nAg and MH groups on the change in ulcer size reduction.

Group	Reference group	Gradient	*p* value
nAg	Conventional	20.537	0.011^*∗*^
MH	Conventional	9.337	0.311
nAg	MH	2.883	0.000^*∗*^

^*∗*^Significant (*p* ≤ 0.05).

**Table 3:** The presence of biofilm at different observation points among the three groups.

Week	Biofilm	Treatment group			*p* value	Significance
		nAg (*n* = 11)	MH (*n* = 10)	Conventional (*n* = 10)		
**1**	Yes	6	6	3	0.439	NS^a^
	No	5	4	7		
	NA^b^	0	0	0		

**2**	Yes	6	6	6	>0.9999	NS^a^
	No	5	4	4		
	NA^b^	0	0	0		

**3**	Yes	9	7	5	0.477	NS^a^
	No	2	3	4		
	NA^b^	0	0	1		

**4**	Yes	7	7	5	0.828	NS^a^
	No	4	3	4		
	NA^b^	0	0	1		

**6**	Yes	4	4	3	>0.9999	NS^a^
	No	5	5	6		
	NA^b^	2	1	1		

**8**	Yes	2	3	2	>0.9999	NS^a^
	No	5	4	4		
	NA^b^	4	3	4		

**10**	Yes	2	2	2	0.879	NS^a^
	No	2	3	4		
	NA^b^	7	5	4		

**12**	Yes	2	3	3	0.271	NS^a^
	No	0	2	3		
	NA^b^	9	5	4		

NS^a^: not significant.

NA^b^: not available (DFU healed).

**Table 4:** Clinical signs of wound infection at different observation points among the three groups.

Week	Infection status	Treatment group			*p* value	Significance
		nAg (*n* = 11)	MH (*n* = 10)	Conventional (*n* = 10)		
**1**	Yes	7	3	6	0.296	NS^a^
	No	4	7	4		
	NA^b^	0	0	0		

**2**	Yes	10	6	9	0.262	NS^a^
	No	1	4	1		
	NA^b^	0	0	0		

**3**	Yes	10	9	8	0.825	NS^a^
	No	1	1	1		
	NA^b^	0	0	1		

**4**	Yes	10	8	9	0.825	NS^a^
	No	1	2	0		
	NA^b^	0	0	1		

**6**	Yes	8	8	9	>0.9999	NS^a^
	No	1	1	0		
	NA^b^	2	1	1		

**8**	Yes	6	7	6	>0.9999	NS^a^
	No	1	0	0		
	NA^b^	4	3	4		

**10**	Yes	3	5	6	0.496	NS^a^
	No	1	0	0		
	NA^b^	7	5	4		

**12**	Yes	2	5	4	0.150	NS^a^
	No	0	0	0		
	NA^b^	9	5	4		

NS^a^: not significant.

NA^b^: not available (DFU healed).

**Table 5:** Gradients of the nAg and MH groups on the change of MMP-9 concentration.

Group	Reference group	Gradient	*p* value
nAg	Conventional	1.263	0.027^*∗*^
MH	Conventional	1.507	0.000^*∗*^
nAg	MH	−0.427	0.098

^*∗*^Significant (*p* ≤ 0.05).

**Table 6:** Adverse event and interventions.

Group	Adverse event	Intervention
nAg	Calf swelling	Oral antibiotics

MH	Generalized blister	Oral steroid and antibiotics prescribed in the family clinic

Conventional	Lung carcinoma	Referred to oncology department and patient discontinued from the study
	Chest infection	Oral antibiotics
	Blisters near the ulcer due to friction on walking	Activity restriction
	Severe wound infection	Admission for intravenous antibiotics and patient discontinued from the study
